# Case Report: Sarcoidosis or tuberculosis? A continuous challenge

**DOI:** 10.3389/fmed.2025.1602370

**Published:** 2025-08-25

**Authors:** Irina Ruxandra Strambu, Ana Beer

**Affiliations:** ^1^Department of Pulmonology, University of Medicine and Pharmacy, Bucharest, Romania; ^2^Department of Pulmonology, Institute of Pneumology, Bucharest, Romania

**Keywords:** sarcoidosis, tuberculosis, *Mycobacterium tuberculosis*, epithelioid granuloma, pathogenesis

## Abstract

Sarcoidosis is a multisystem granulomatous disorder of unknown etiology, characterized by the formation of non-caseating granulomas in affected tissues and organs. In over half of the cases, the disease undergoes spontaneous remission. In contrast, tuberculosis (TB) is an infectious disease caused by *Mycobacterium tuberculosis*, which, if left untreated, can be fatal. Sarcoidosis and tuberculosis exhibit numerous overlapping clinical, radiological, and histopathological features, including the presence of epithelioid cell granulomas with multinucleated giant cells. Historically, a potential etiological role of *M. tuberculosis* in sarcoidosis has been proposed; however, this hypothesis has not been conclusively supported by current evidence or therapeutic outcomes. Differentiating between these two entities presents a significant diagnostic challenge, particularly in regions with a high prevalence of tuberculosis. The diagnostic complexity is further heightened in cases where a concomitant occurrence of both conditions is suspected. In such scenarios, the absence of a definitive biomarker hampers the ability to discern whether the diseases coexist independently or share a pathogenic link. This article reviews current evidence on the association between sarcoidosis and tuberculosis and explores potential pathways to elucidate their etiological interrelationship.

## Introduction

Sarcoidosis is a systemic granulomatous disorder of unknown etiology, characterized by the formation of non-caseating epithelioid cell granulomas in various organs. Thoracic involvement is observed in over 90% of cases, typically affecting the mediastinal lymph nodes and pulmonary parenchyma (1). Histologically, sarcoid granulomas are composed of tightly clustered epithelioid histiocytes, multinucleated giant cells, and lymphocytes, with minimal or absent central fibrinoid necrosis (2). The identification of granulomas on histopathological examination remains a cornerstone in the diagnostic evaluation of sarcoidosis, with earlier diagnostic guidelines recommending biopsy-proven granulomas as a prerequisite for a definitive diagnosis (3).

Although epithelioid granulomas are part of the definition of sarcoidosis, similar histopathological patterns are observed in a broad spectrum of other conditions—including infectious, inflammatory, and neoplastic diseases—rendering differential diagnosis a complex task (4). Among these, tuberculosis (TB) represents the most critical differential diagnosis, as it is also characterized by granulomatous inflammation. The granulomas in TB, however, tend to demonstrate more pronounced central necrosis. Despite their morphological similarities under the microscope, sarcoidosis and tuberculosis differ substantially in terms of etiology, therapeutic approach, and prognosis.

Beyond histopathological overlap, sarcoidosis and TB share numerous clinical manifestations and radiological features, further complicating the diagnostic process.

Tuberculosis, caused by *Mycobacterium tuberculosis* (*M. tuberculosis*), is the second leading cause of death from infectious diseases in adults worldwide, following HIV. Humans serve as the natural reservoir for this aerobic, intracellular bacillus, which primarily replicates within macrophages. Infection with *M. tuberculosis* triggers a robust immune response characterized by cytokine and chemokine release, leading to the recruitment of monocytes, macrophages, and neutrophils and ultimately to the formation of granulomas—known as tubercles—with central caseous necrosis. *M. tuberculosis* may remain dormant, leading to latent TB infection, or it may progress to active disease. Latent TB is characterized by an immunological response to mycobacterial antigens, detectable through tuberculin skin testing, without clinical or radiological evidence of active disease (5).

Distinguishing between sarcoidosis and TB is particularly challenging in regions with a high burden of tuberculosis, such as India, China, South Africa, Romania, and Moldova. In these settings, sarcoidosis may be underdiagnosed or misclassified and treated as TB due to the overlapping clinical and imaging features and the high endemicity of tuberculosis (6, 7).

From an etiopathogenic standpoint, it is important to recall that the underlying cause of sarcoidosis remains elusive. Several hypotheses have been proposed, implicating environmental exposures, bacterial infections, and autoimmune mechanisms. Among the microbial triggers, mycobacteria have been suggested as potential etiological agents, raising the possibility of an intrinsic link between sarcoidosis and TB pathogenesis. Therefore, beyond diagnostic differentiation, the hypothesis that mycobacteria could be the cause of sarcoidosis warrants reconsideration.

The distinction between the two diseases has significant therapeutic implications. Sarcoidosis is typically managed with corticosteroids or immunosuppressants, while TB requires prolonged antimicrobial therapy. Misdiagnosis may result in inappropriate corticosteroid administration in undiagnosed TB patients, potentially leading to disease exacerbation and dissemination. In contrast, TB typically responds rapidly to specific anti-tubercular therapy, with symptomatic and radiologic improvement observed within the first two to 3 months of treatment. Sarcoidosis may exhibit spontaneous resolution of symptoms and imaging changes within a few months. When treatment is considered necessary, a prompt clinical and radiological response is often observed within the first month of corticosteroid therapy. Consequently, initial diagnosis must always be thorough. In ambiguous cases, standard practice may involve initiating empirical anti-TB treatment, followed by reassessment after approximately 3 months. Simultaneous administration of both anti-tubercular drugs and corticosteroids may improve symptoms but complicates the interpretation of therapeutic efficacy.

This article aims to explore the diagnostic challenges in distinguishing sarcoidosis from tuberculosis and to examine the possible etiological relationship between these two granulomatous conditions.

## A complicated case: diagnostic challenge between sarcoidosis and tuberculosis

**Figure 1:**
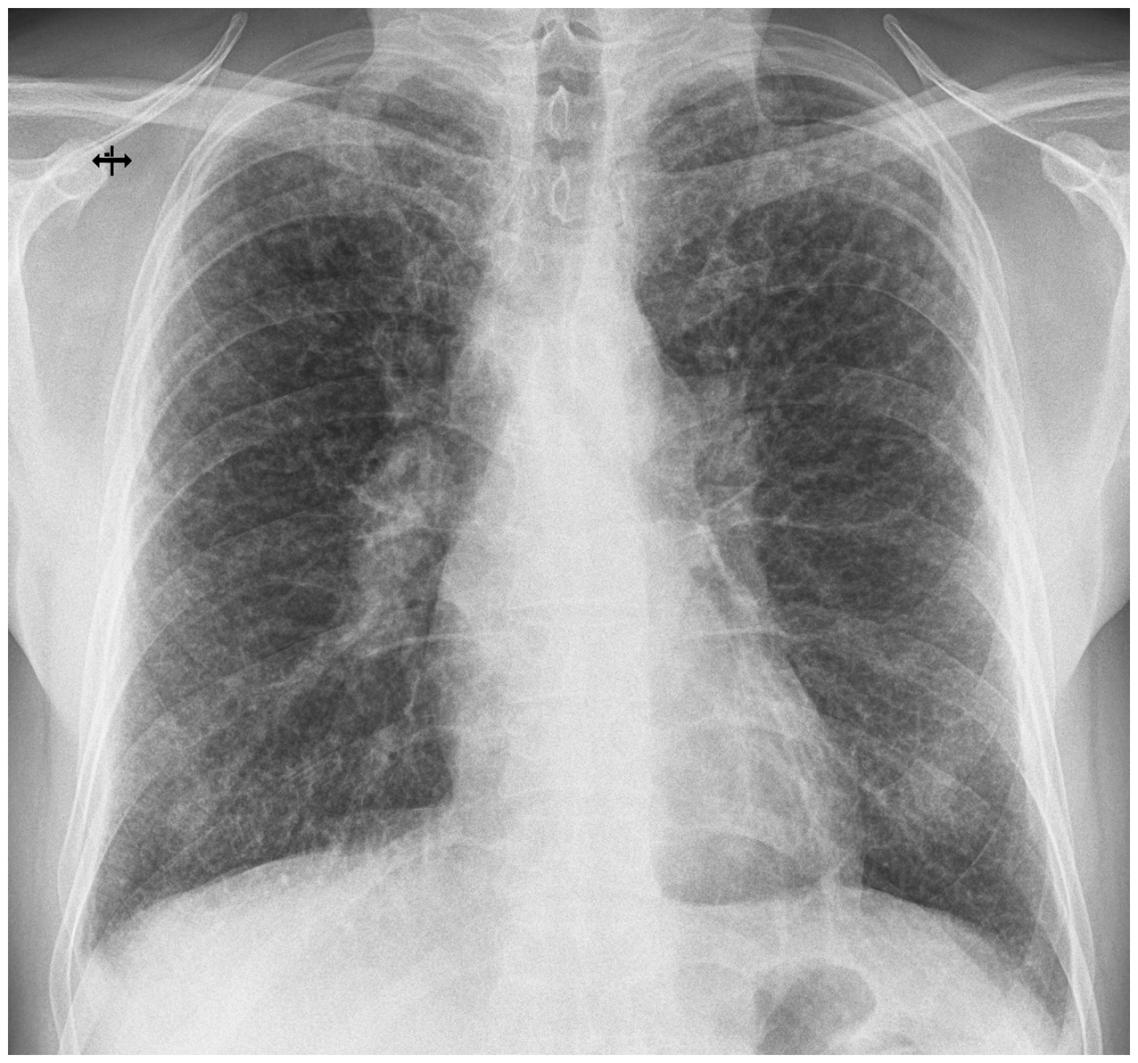
Postero-anterior chest X-ray at presentation: multiple ill-defined, low-intensity micronodules distributed bilateral, with upper lobe predominance.

**Figure 2:**
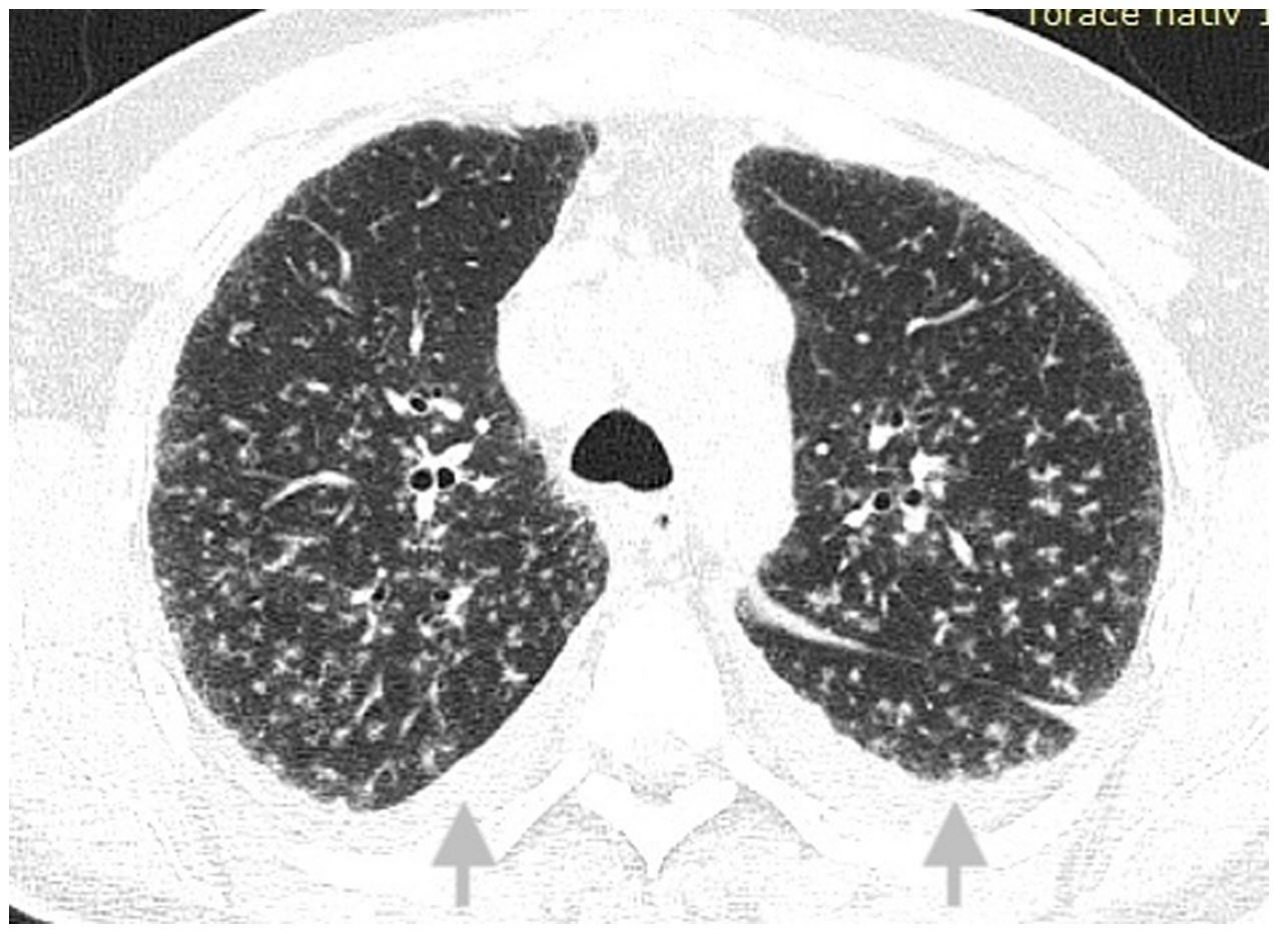
CT scan of the thorax at initial presentation, lung window: multiple nodules with perilymphatic distribution and minor bilateral pleural effusions (arrows).

**Figure 3:**
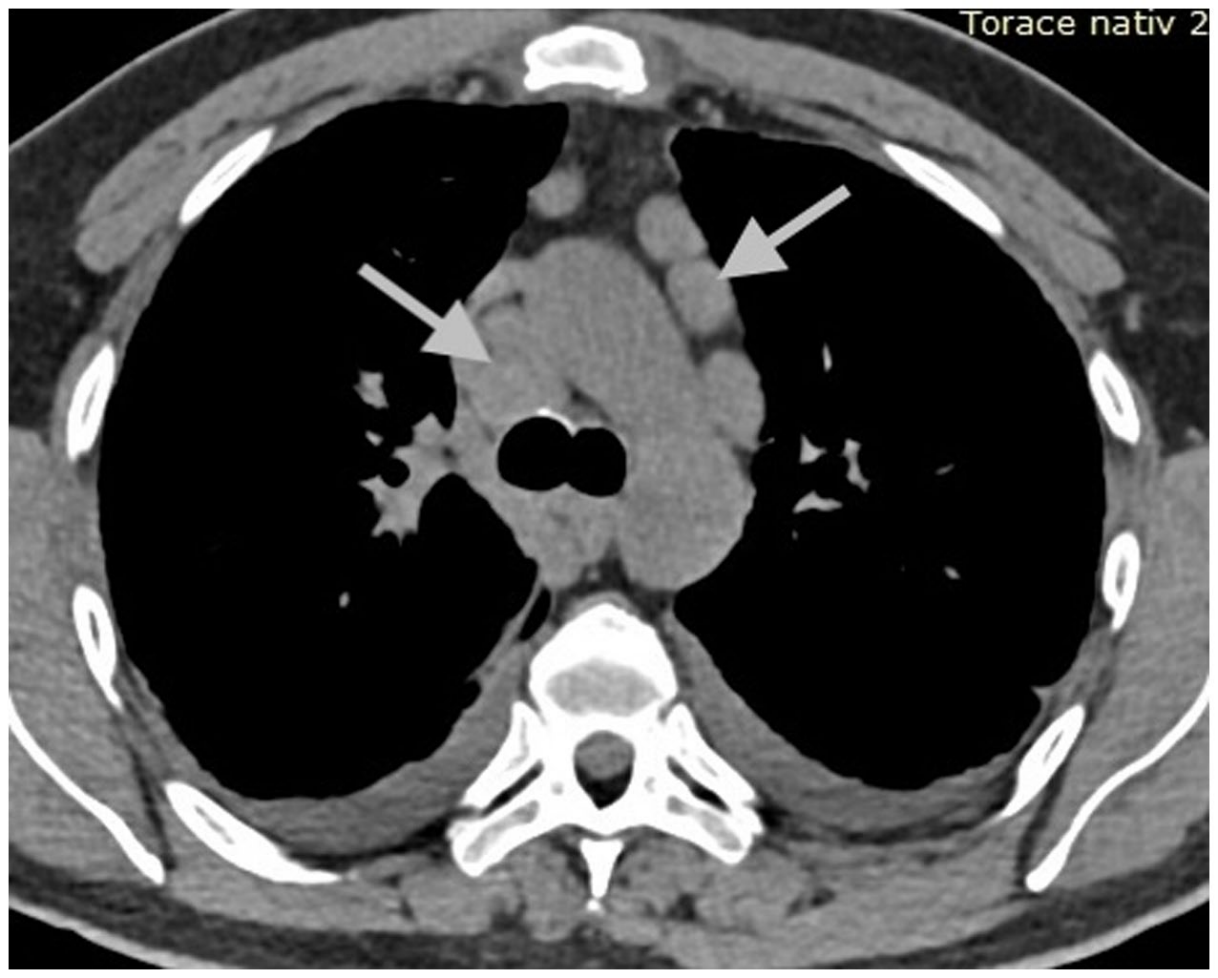
CT scan of the thorax at initial presentation, mediastinal window: enlarged mediastinal lymph nodes (arrows).

To best illustrate the complexity of the sarcoidosis–tuberculosis diagnostic spectrum, we present the case of a 48-year-old Caucasian male individual admitted with constitutional symptoms, including significant weight loss (10 kg), non-productive cough, and mild exertional dyspnea, with an insidious onset approximately 3 months prior to admission. The patient had no significant professional exposure (he was a taxi driver) and had no recollection of any previous contact with TB patients in his family and professional environment. He had no family history of sarcoidosis, other granulomatosis, or TB. Clinical examination at presentation showed no abnormal lung sounds. No other abnormalities were found, including absence of fever, peripheral lymph node enlargement, or findings related to the eye, heart, or abdomen. Chest X-ray showed multiple micronodules distributed especially in the upper regions of the lungs (Figure 1). High-resolution chest computed tomography (CT) confirmed multiple bilateral pulmonary nodules, predominantly in the upper lobes, exhibiting a perilymphatic distribution. These findings were associated with mediastinal lymphadenopathy and minimal bilateral pleural effusion (Figures 2, 3).

Initial laboratory investigations indicated systemic inflammation, with a mildly elevated serum angiotensin-converting enzyme (ACE) level, hypercalcemia, and increased urinary calcium excretion. Bronchoscopy with bronchoalveolar lavage (BAL) demonstrated lymphocytic alveolitis without cytological evidence of malignancy. Acid-fast bacilli (AFB) were not identified on direct smear or molecular testing (polymerase chain reaction - PCR) of BAL samples, and mycobacterial cultures remained negative after 2 months. Pulmonary function testing showed a moderate restrictive ventilatory defect and a parallel reduction in diffusing capacity for carbon monoxide (DLCO).

Based on the clinical, radiological, and BAL findings, pulmonary sarcoidosis was considered the leading diagnosis. However, given the high prevalence of tuberculosis (TB) in Romania and the overlapping features with sarcoidosis, TB could not be definitively excluded. Notably, paucibacillary TB lesions, such as nodules or infiltrates, may yield false-negative results on smear and culture tests. Unlike cavitary lesions, where mycobacteria thrive and multiply by millions, these “closed” lesions—such as nodules, infiltrates, and lymph node involvement—may harbor small quantities of mycobacteria, insufficient for detection in respiratory secretions.

A definitive diagnosis was deemed necessary for choosing the correct treatment. Therefore, the patient underwent a video-assisted thoracoscopic surgery (VATS) lung biopsy 1 month after admission. As a precaution, empirical anti-TB therapy (isoniazid, rifampin, pyrazinamide, and ethambutol) was initiated preoperatively, 1 month before the operation. This is a common precaution for patients suspected of tuberculosis undergoing surgical procedures, with the intention to prevent potential hematogenous dissemination of *Mycobacterium tuberculosis* during surgery. Dissemination triggered by surgical procedures has been documented in several case reports (8–10).

Histopathological examination of the lung biopsy specimens revealed well-formed epithelioid granulomas with minimal central necrosis. Ziehl–Neelsen staining, performed in the tissue already processed and embedded in paraphyn, unexpectedly identified AFB within the granulomas. Mycobacterial culture was not feasible at this stage. While caseating necrosis is typically expected in TB, the granulomas in this case bore closer histological resemblance to those of sarcoidosis. However, the presence of AFB led to a working diagnosis of tuberculosis. It is important to note that pathologists often have a hard time differentiating sarcoid granulomas from TB granulomas. The main difference lies in the amount of necrosis present in the center of the granuloma. Sarcoidosis is associated with little or no necrosis, while in TB, necrosis with caseous formation is quite common.

In our patient, the anti-TB regimen was continued, given the potential harm of initiating corticosteroid therapy in a patient with active TB. A positive response to TB treatment would typically be expected within 2 to 3 months, including improvement of dyspnea, recovery of appetite, and signs of resolution of changes on CT scan. As a general rule, if both treatments are necessary, it is recommended to start anti-TB treatment at least 1 month before initiating immunosuppressive treatment, to avoid potential exacerbation of TB, if this exists and is active.

**Figure 4:**
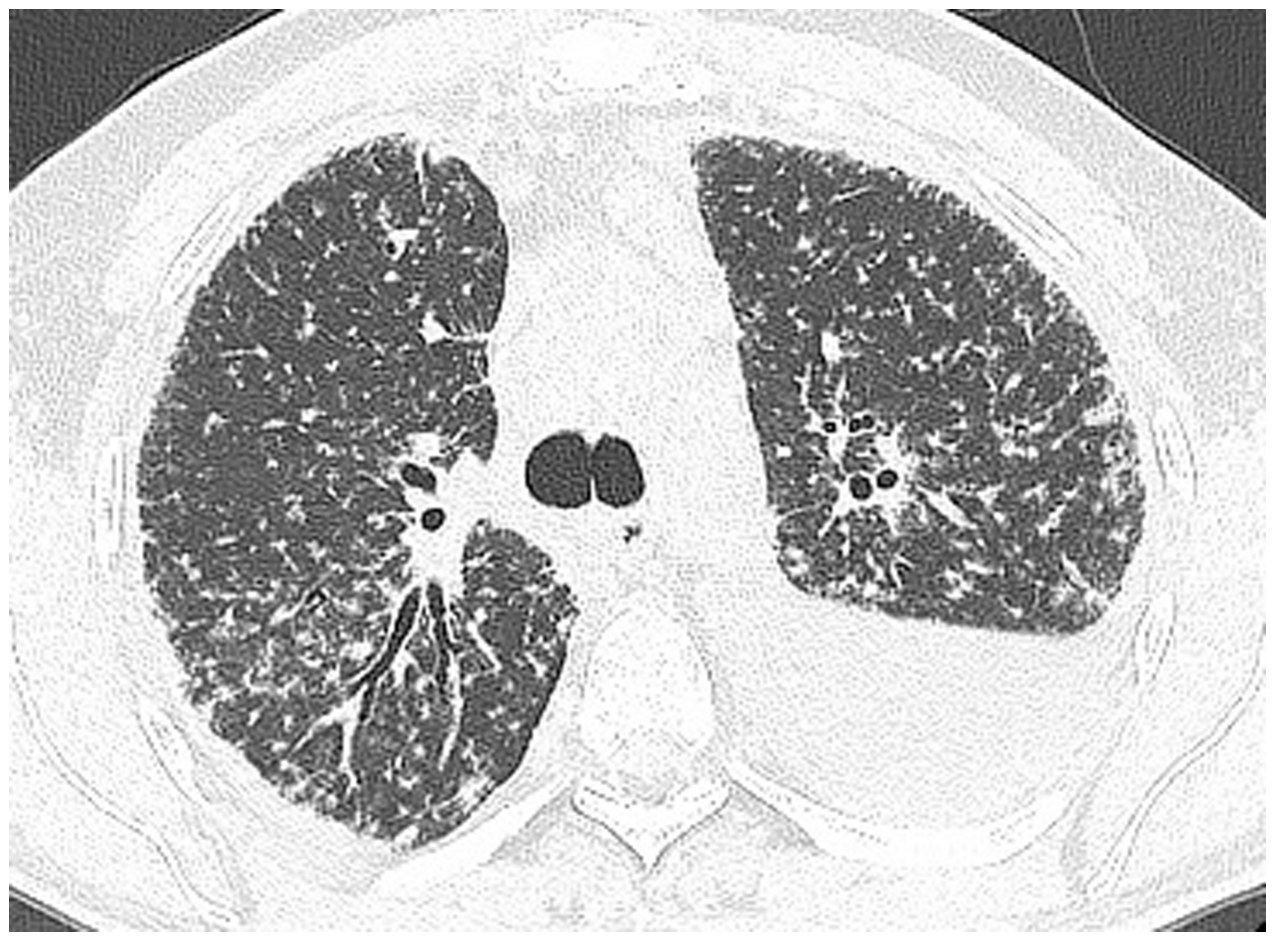
CT scan after 3 months of anti-tuberculosis treatment: persistent lung nodules embedded in a fine network, and a significant increase in left pleural effusion, compared to the initial scan.

Despite appropriate anti-TB treatment, the patient showed no clinical or radiographic improvement over a three-month follow-up period. Cough, fatigue, and low appetite persisted. He had no hemoptysis and no night sweats. Serum ACE levels increased further, and CT imaging demonstrated worsening of pulmonary infiltrates and increased left-sided pleural effusion. The pulmonary nodules evolved, acquiring a more solid appearance and becoming incorporated into a reticular network (Figure 4). Thoracentesis yielded an exudate with an adenosine deaminase (ADA) level of 33 U/L, below the 40 U/L threshold typically indicative of TB (11). The ADA level in the pleural fluid argued against the tuberculosis origin of the pleurisy. The pleural fluid was negative for AFB on smear, culture, and molecular testing. Cytology confirmed a lymphocyte-predominant effusion without malignant cells.

After 4 months of anti-TB therapy and no clinical or radiological improvement, a revised diagnosis was considered—either sarcoidosis alone or concomitant sarcoidosis and TB. Methylprednisolone 32 mg/day was initiated while maintaining anti-TB therapy. Within 2 months, the patient experienced marked symptomatic improvement: resolution of cough, weight gain, and diminished dyspnea. However, pulmonary function tests showed only minimal improvement, and radiological changes persisted.

**Figure 5:**
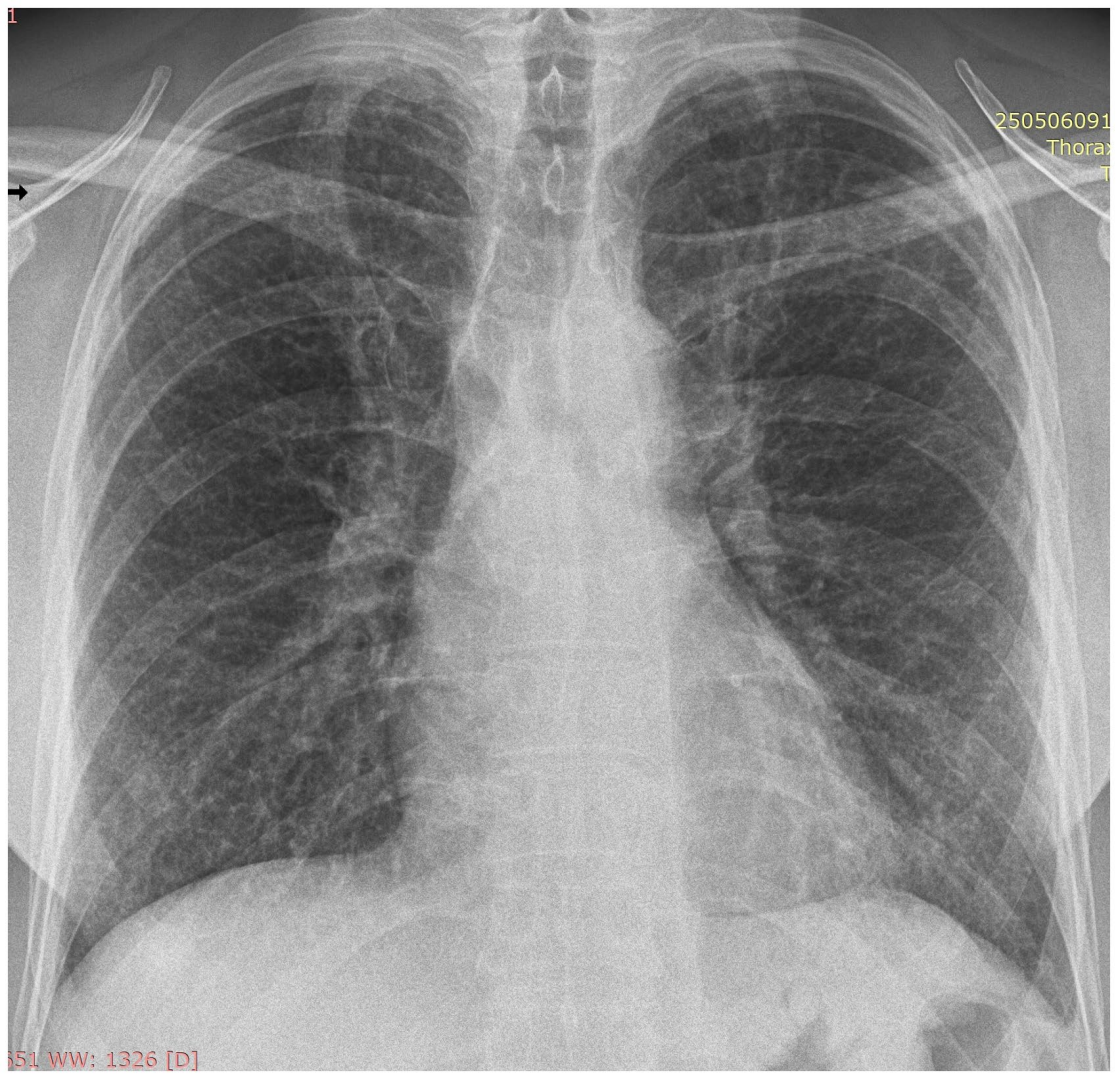
Postero-anterior chest X-ray performed 6 months after starting oral corticosteroids: no pleural effusion and significant resorption of lung nodules.

Anti-TB therapy was discontinued after 6 months, having fulfilled the standard treatment duration. The anti-TB regimen was maintained after the initiation of oral corticosteroids as the patient experienced no side effects, and once initiated, the treatment was carried on to its completion. Corticosteroids were continued in a tapered regimen, planned for at least 12 months. Follow-up at 6 months after steroid initiation (and 4 months after cessation of anti-TB therapy) revealed continued clinical improvement, with resolution of respiratory symptoms and significant weight gain. A chest X-ray demonstrated near-complete radiological resolution, including disappearance of the pleural effusion and substantial regression of interstitial nodules (Figure 5).

Several diagnostic scenarios were considered. The absence of improvement during anti-TB treatment and the favorable response to corticosteroids strongly suggest that sarcoidosis was the primary disease process, and this is the final diagnosis we considered for this patient. Alternatively, the patient may have harbored an infection with non-tuberculous mycobacteria, which are less responsive to standard TB regimens; however, this hypothesis lacks microbiological confirmation and is not consistent with the improvement observed after starting corticosteroid treatment. A further possibility is the coexistence of both sarcoidosis and TB, with initial mycobacterial infection triggering a granulomatous immune response akin to sarcoidosis—a phenomenon previously described in the literature. This would be consistent with the sequential treatment of both diseases leading to a positive outcome. Lastly, another granulomatous disease cannot be entirely ruled out, although the clinical response to corticosteroids supports the diagnosis of sarcoidosis.

## Many questions, few answers

This case illustrates the complex and often controversial interplay between sarcoidosis and tuberculosis (or mycobacterial infections in general), raising several unresolved questions:

- Can sarcoidosis and active tuberculosis (as active disease) co-exist in the same patient?
- Can *Mycobacterium tuberculosis* act as a causative agent of sarcoidosis? Could a “sarcoid-like” reaction be mounted in response to active TB?
- Is it possible for *M. tuberculosis* to trigger a sarcoid reaction during both latent and active stages of infection?
- Could non-tuberculous mycobacteria (NTM) be implicated in the pathogenesis of sarcoidosis?
- Could the identification of acid-fast bacilli (AFB) in tissue samples reflect a prior, now inactive TB infection, rather than an active disease process?
- Could TB emerge as an infectious complication of immunosuppressive therapy for sarcoidosis?

None of these questions has a definitive answer. The two conditions share striking similarities in clinical presentation, radiologic appearance, and histopathological findings—most notably, the presence of non-caseating epithelioid granulomas. Both diseases appear capable of eliciting comparable immune responses (12). Some authors have proposed that TB and sarcoidosis represent two ends of a pathophysiologic spectrum, advocating a classification that includes sarcoidosis (S), sarcoid-tuberculous (ST), tuberculous sarcoid (TS), and tuberculosis (TB) (12). However, this hypothesis has yet to gain widespread scientific or clinical validation.

The diagnostic overlap between sarcoidosis and tuberculosis poses significant challenges, especially in countries with a high prevalence of TB. This issue has been the subject of several comprehensive reviews (6, 13, 14).

### Can sarcoidosis and tuberculosis coexist in the same patient?

Numerous case reports support the co-occurrence of sarcoidosis and tuberculosis, particularly in patients from regions with high TB incidence, such as India, China, and Brazil. These reports describe patients with diverse clinical manifestations (e.g., respiratory, dermatologic, gastrointestinal), who meet histological criteria for sarcoidosis while also testing positive for *M. tuberculosis* by molecular methods such as PCR from BAL fluid or tissue biopsies. In most of these cases, granulomatous inflammation is interpreted as evidence of sarcoidosis, although histologic differentiation from TB granulomas remains difficult.

Many such patients received both anti-tubercular therapy (ATT) and corticosteroids—often concurrently—with clinical improvement that could not be definitively attributed to one treatment over the other. Laboratory findings characteristic of sarcoidosis, including elevated ACE levels, hypercalcemia, and systemic symptoms such as fatigue, were frequently observed—paralleling the case we described (15–17).

The prevalence of TB and sarcoidosis coexistence is probably low, coming as a surprise during the diagnostic process. There are no guidelines on how to approach this combination, but it is most likely probable that patients will receive anti-TB treatment for confirmed tuberculosis, while sarcoidosis may or may not be treated, considering the frequent spontaneous resolution of the disease.

Collectively, these reports do not provide definitive answers to our questions but rather reinforce the concept that both sarcoidosis and TB are heterogeneous, chameleonic diseases with an unmatched variety of clinical manifestations.

### Are mycobacteria present in patients with sarcoidosis?

The potential association between sarcoidosis and mycobacteria has long been a subject of investigation. The identification of mycobacteria in patients with sarcoidosis appears to be relatively common.

A meta-analysis spanning over two decades (1980–2006) reviewed 31 studies employing molecular methods, predominantly polymerase chain reaction (PCR), to detect mycobacterial DNA in biological samples (blood, BAL, and tissue biopsies) from patients with sarcoidosis. Among the 874 patients analyzed, 231 tested positive for mycobacterial DNA, with a positive signal rate of 26.4 (23.6–29.5%). The pooled odds ratio for detecting mycobacteria in sarcoidosis samples versus controls was 9.67 (random effects model) and 19.49 (exact method) (18). The authors concluded that the study supported the consistent presence of mycobacteria in sarcoidosis lesions. These findings support a potential role for mycobacteria—particularly *M. tuberculosis*—in the pathogenesis of sarcoidosis in a subset of patients (18).

Conversely, other studies have failed to confirm this association. For instance, analyses of endobronchial ultrasound (EBUS)-guided lymph node biopsies from patients with either sarcoidosis or malignancy showed no significant difference in the identification of mycobacterial agents, with no mycobacteria detected in either group (19).

PCR, while highly sensitive and capable of detecting minute fragments of mycobacterial DNA, cannot distinguish between active infection, latent TB, or residual DNA from previously treated or self-resolving infections. In high TB burden settings such as India, this limitation significantly reduces its diagnostic utility. For this reason, the use of PCR to differentiate sarcoidosis from TB is not recommended in endemic regions (6).

### Is there serological evidence of mycobacterial involvement in sarcoidosis?

Beyond PCR, several studies have explored serological evidence of immune responses to mycobacterial antigens in sarcoidosis. As early as 1964, a study reported that nearly 80% of 280 patients with sarcoidosis exhibited serum reactivity to mycobacterial antigens (20).

A more recent meta-analysis (2016) assessed immunologic evidence of mycobacterial involvement, including both humoral and cell-mediated responses. The analysis included 13 case–control studies and 733 participants. Of the nine trials evaluating T-cell responses to *M. tuberculosis* antigens, 126 of 253 sarcoidosis samples tested positive—significantly more than the controls (*p* < 0.00001). Similarly, in four studies analyzing humoral responses, 55 of 100 patients with sarcoidosis showed seropositivity, again significantly higher than the control group (*p* < 0.00001) (5). These findings suggest that prior or ongoing exposure to mycobacterial antigens may play a role in sarcoidosis pathogenesis.

### Could viable mycobacteria be isolated from sarcoid tissue?

From a microbiological standpoint, the strongest evidence of mycobacterial pathogenicity in sarcoidosis would be the culture of viable organisms from affected tissues. However, efforts to culture mycobacteria from sarcoid lesions have largely been unsuccessful. One previous study that employed prolonged culture techniques (up to 12 months) failed to isolate *M. tuberculosis* from the mediastinal lymph nodes of patients with histologically confirmed sarcoidosis (21). These findings argue against a direct etiological role for mycobacteria in most cases of sarcoidosis.

### Could mycobacteria be the cause of sarcoidosis?

The hypothesis that *Mycobacterium tuberculosis* may contribute to the etiology of sarcoidosis has a longstanding history. In 1960, Scadding published a seminal study evaluating 230 histologically confirmed sarcoidosis cases. In 29 patients (13%), *Mycobacterium tuberculosis* (“tubercle bacilli”) was identified at some point during their clinical course—prior to, concurrent with, or following the diagnosis of sarcoidosis. Notably, in 18 patients, *M. tuberculosis* was detected in the absence of clinical or radiological evidence of active tuberculosis. However, the identification of *M. tuberculosis* in these cases was based on samples obtained from sputum, gastric aspirate, or postmortem tissue cultures, rather than directly from sarcoid lesions. Importantly, no significant therapeutic response to anti-tuberculous agents was observed. In Scadding’s opinion, all cases with identified mycobacteria represented tuberculosis with a “sarcoid phase” evolving into “overt tuberculosis phase,” although the lack of response to therapy remained unexplained. Notably, at that time, antimicrobial resistance had not yet been recognized as a clinical phenomenon, and tuberculosis prevalence was high in Western Europe (22).

A more contemporary approach to exploring the potential etiologic link between *M. tuberculosis* and sarcoidosis involves evaluating the effect of anti-tuberculous therapy in patients with sarcoidosis. A randomized, placebo-controlled phase II clinical trial published in 2021 assessed the efficacy of an 8-week anti-tuberculous regimen (CLEAR therapy) in patients with progressive pulmonary sarcoidosis. The study evaluated changes in forced vital capacity (FVC) and six-minute walk test (6MWT) performance. No significant differences were observed between the treatment and placebo groups in either outcome. In addition, no improvement was reported in quality-of-life metrics, as assessed by the St. George’s Respiratory Questionnaire (23).

### Could other mycobacteria play a role in sarcoidosis pathogenesis?

*Mycobacterium avium* subspecies *paratuberculosis* (MAP), historically linked to Crohn’s disease, has also been implicated in a range of immune-mediated diseases, including Blau syndrome, autoimmune (Hashimoto’s) thyroiditis, type 1 diabetes, multiple sclerosis, systemic lupus erythematosus, rheumatoid arthritis, and Parkinson’s disease. MAP, a zoonotic pathogen commonly found in ruminants, can persist in environmental reservoirs such as water and soil for extended periods (up to 120 weeks), representing a potential environmental exposure risk. It has been hypothesized that MAP may be involved in the pathogenesis of sarcoidosis, potentially mediated by shared genetic susceptibility factors with mycobacterial infection (24).

Early serological evidence supporting a role for MAP in sarcoidosis was reported by Chiodini in 1993, where patients with sarcoidosis exhibited antibody reactivity against MAP antigens (25). Further reports have documented the detection of MAP in sarcoid tissue—particularly in younger patients—and have described successful treatment of cardiac sarcoidosis using anti-MAP antibiotic regimens (26). In the United States, MAP infection is prevalent in livestock, with estimated seroprevalence rates as high as 80%, raising concerns regarding zoonotic transmission and its possible role in human granulomatous disease. By contrast, a veterinary study conducted in Romania found significantly lower MAP seroprevalence in cattle (6.25%), sheep (19.54%), and goats (28.2%) (27). Given the higher prevalence of *M. tuberculosis* infection in Romania, it may represent a more plausible candidate in the regional pathogenesis of sarcoidosis.

### Sarcoidosis mimicking tuberculosis, and vice versa

Sarcoidosis can closely mimic tuberculosis in clinical presentation, imaging, and histopathology. In patients residing in TB-endemic areas or with known TB exposure, tuberculosis is often prioritized as the initial diagnosis. Certain radiologic findings, such as the “galaxy sign,” may favor sarcoidosis, but these features may not be evident early in the disease course (28). Conversely, tuberculosis may also mimic sarcoidosis. Cases initially presumed to be sarcoidosis have subsequently been confirmed as tuberculosis following molecular diagnostics (29).

### Can we differentiate between sarcoidosis and tuberculosis?

Given the divergent treatment pathways, distinguishing between sarcoidosis and tuberculosis is critical. A fundamental step in the diagnostic algorithm for suspected sarcoidosis is the exclusion of active pulmonary TB (30, 31).

- Tuberculin skin test (TST): The test is typically positive in TB and negative in sarcoidosis. However, its diagnostic value is reduced in TB-endemic regions and among populations with widespread Bacillus Calmette-Guérin (BCG) vaccination. TST negativity in sarcoidosis has a reported negative predictive value of 86% (32). The “immune paradox,” wherein lymphocytes are sequestered in granulomas at sites of intense inflammation, resulting in peripheral anergy, could lead to a negative TST in sarcoidosis patients, irrespective of their previous TB status.
- Interferon-gamma release assays (IGRAs): These assays offer improved specificity compared to the TST, but they cannot distinguish between latent TB and sarcoidosis (32).
- Bronchoalveolar lavage (BAL) CD4/CD8 ratio: An elevated CD4/CD8 ratio in BAL fluid is suggestive of sarcoidosis. Nonetheless, a meta-analysis reported a positive predictive value of only 70% (33), and another meta-analysis demonstrated that the ratio can also be elevated in TB (34).
- PET-CT imaging: Fluorodeoxyglucose (FDG)-PET is valuable in assessing disease activity and guiding biopsy, but it lacks specificity due to FDG uptake in both tuberculous and sarcoid lesions (35).
- Transcriptomic analysis: Whole-blood transcriptomic profiling has shown potential for distinguishing between sarcoidosis and TB, with disease-specific patterns of gene expression, including differential regulation of interferon-inducible genes (36). However, this remains a research tool with limited clinical applicability.
- Sarcoidosis diagnostic score (SDS): Developed as the SDS-Biopsy and SDS-Clinical variants, this scoring system can help differentiate sarcoidosis from infectious and non-infectious mimickers. While the tool demonstrated acceptable performance, its discriminative power decreased in male patients and TB-endemic regions. The SDS also struggled to differentiate sarcoidosis from other granulomatous disorders such as chronic beryllium disease (37, 38).

## Future directions

In clinical practice, the exclusion of tuberculosis remains the most critical step in diagnosing sarcoidosis. In most cases, the proof of the presence of active mycobacteria in accessible samples (such as sputum or BAL) combined with a positive response to treatment allows for accurate differentiation between tuberculosis patients and patients with sarcoidosis. However, diagnostic uncertainty persists in a subset of patients where the distinction between sarcoidosis and TB—or a sarcoid-like reaction to dormant or active mycobacteria—remains ambiguous.

How can we progress in trying to answer all these questions? Are these questions worth answering? Would the answers benefit patient care or deepen our understanding of sarcoidosis pathogenesis?

Several research directions may help elucidate this relationship:

- Culturing viable mycobacteria from sarcoid tissue, rather than relying solely on molecular techniques.
- Identifying granuloma-specific biomarkers capable of differentiating between sarcoid and tuberculous granulomas.
- Routine screening for latent TB infection in patients with newly diagnosed sarcoidosis.
- Epidemiological studies to determine sarcoidosis prevalence in TB-endemic regions, which could help understand disease overlap and potential pathophysiological connections.

Finally, the possibility that sarcoidosis represents, in a subset of patients, an immunologic response to latent mycobacterial antigens remains plausible. Further research should investigate whether such cases are more symptomatic and require treatment, or if they follow a benign, self-limited course typical of many sarcoidosis presentations.

## Data Availability

The original contributions presented in the study are included in the article/supplementary material, further inquiries can be directed to the corresponding author/s.
